# Increased Cathepsin S activity associated with decreased protease inhibitory capacity contributes to altered tear proteins in Sjögren’s Syndrome patients

**DOI:** 10.1038/s41598-018-29411-9

**Published:** 2018-07-23

**Authors:** Maria C. Edman, Srikanth R. Janga, Zhen Meng, Mercy Bechtold, Alexander F. Chen, Chongiin Kim, Luke Naman, Arunava Sarma, Neha Teekappanavar, Alice Y. Kim, Sara Madrigal, Simranjit Singh, Elizabeth Ortiz, Stratos Christianakis, Daniel G. Arkfeld, Wendy J. Mack, Martin Heur, William Stohl, Sarah F. Hamm-Alvarez

**Affiliations:** 1 Department of Ophthalmology, USC Roski Eye Institute, Keck School of Medicine, University of Southern California, Los Angeles, CA USA; 2 Department of Pharmacology and Pharmaceutical Sciences, School of Pharmacy, Los Angeles, University of Southern California, Los Angeles, CA USA; 3 Keck School of Medicine, University of Southern California, Los Angeles, CA USA; 4 Division of Rheumatology, Department of Medicine, Keck School of Medicine, University of Southern California, Los Angeles, CA USA; 5 Department of Preventive Medicine, Keck School of Medicine, University of Southern California, Los Angeles, CA USA; 6 Division of Rheumatology, Department of Medicine, Los Angeles County + University of Southern California Medical Center, Los Angeles, CA USA

## Abstract

Cathepsin S (CTSS) activity is elevated in Sjögren’s Syndrome (SS) patient tears. Here we tested whether protease inhibition and cystatin C (Cys C) levels are reduced in SS tears, which could lead to enhanced CTSS-driven degradation of tear proteins. CTSS activity against Cys C, LF and sIgA was tested in SS or healthy control tears. Tears from 156 female subjects (33, SS; 33, rheumatoid arthritis; 31, other autoimmune diseases; 35, non-autoimmune dry eye (DE); 24, healthy controls) were analyzed for CTSS activity and Cys C, LF, and sIgA levels. Cys C and LF showed enhanced degradation in SS tears supplemented with recombinant CTSS, but not supplemented healthy control tears. CTSS activity was significantly increased, while Cys C, LF and sIgA levels were significantly decreased, in SS tears compared to other groups. While tear CTSS activity remained the strongest discriminator of SS in autoimmune populations, combining LF and CTSS improved discrimination of SS beyond CTSS in DE patients. Reductions in Cys C and other endogenous proteases may enhance CTSS activity in SS tears. Tear CTSS activity is reconfirmed as a putative biomarker of SS in an independent patient cohort while combined LF and CTSS measurements may distinguish SS from DE patients.

## Introduction

Sjögren’s syndrome (SS) is a systemic autoimmune disease characterised by lymphocytic infiltration and loss of secretory function of lacrimal glands (LG) and salivary glands. In addition to keratoconjunctivitis sicca and xerostomia, constitutional symptoms are common, and may involve pulmonary, neurological, vascular, and renal systems^1^. However, onset of sicca and systemic symptoms is often not parallel, making early diagnosis a challenge in patients presenting primarily with either dry eye or systemic symptoms^2,3^.

The lack of specificity of currently-used biomarkers combined with the invasive nature of minor salivary gland biopsies have prompted efforts to identify new non-invasive biomarkers for diagnosis of SS. Tear and salivary biomarkers may be especially relevant, since alterations in the composition of these fluids may mirror the pathological processes and functional status of these glands affected by SS.

Tear cathepsin S (CTSS) activity may be one such biomarker. CTSS activity in tears of male NOD mice, a murine model of SS, is greater than that in tears of healthy BALB/c mice^4^, while CTSS activity in tears of SS patients is greater than that in tears of patients with rheumatoid arthritis (RA), systemic lupus erythematosus (SLE), non-autoimmune dry eye (DE) disorders, and healthy controls^5^. CTSS is an intriguing protein with functions linked to inflammation including regulation of major histocompatibility complex class II-mediated antigen presentation^6,7^. Furthermore, it also has important functions in extracellular matrix degradation^8^ and activation of the protease activated receptor 2^9^ which mediates inflammatory pain^10,11^, triggering cytokine production and itchiness^12^. Moreover, since CTSS exhibits potent activity at both acid and neutral pH levels^13^, elevated tear CTSS activity may alter tear composition, thereby affecting the ocular surface. Indeed, proteoglygan 4/lubricin, a glycoprotein important for lubrication and smooth movement of the eyelids, is degraded by tear CTSS^14^.

The activities of cysteine cathepsins, including CTSS, are physiologically regulated by a family of protease inhibitors, cystatins^15^. Cystatin C (Cys C) is the most abundant and potent inhibitor of CTSS, and changes in its levels in the extracellular environment are implicated in formation of atherosclerotic plaques and tumor metastases^16–18^. Accordingly, changes in tear Cys C levels could affect tear CTSS activity.

In this study, we hypothesized that tears of SS patients may contain reduced levels of Cys C, and possibly other endogenous protease inhibitors as well, thereby allowing tear CTSS to directly or indirectly enhance the degradation of other tear proteins. We demonstrate that reduced Cys C tear levels are correlated with increased CTSS activity in LG and tears of SS-model mice and in tears of SS patients. Furthermore, we demonstrate that CTSS, at activity levels found in tears of SS patients but not in tears of healthy controls, promotes degradation of two other abundant tear proteins, lactoferrin (LF) and secretory IgA (sIgA), both of which play fundamental roles in ocular defense against pathogens^19–21^. Finally, we demonstrate remarkably reduced levels of Cys C, LF, and sIgA in combination with elevated CTSS activity in SS patient tears relative to levels in tears from patients with other rheumatic diseases, non-autoimmune DE, and healthy controls. Intriguingly, while these decreases do not offer the ability to further discriminate SS within the autoimmune disease population beyond that of CTSS activity, the combination of LF and CTSS measurements may allow better discrimination between those individuals with SS and those with non-autoimmune DE, thereby potentially offering a new solution to an ongoing clinical challenge.

## Results

### Cystatin C is reduced in tears and LG of male NOD mice

**Figure 1:**
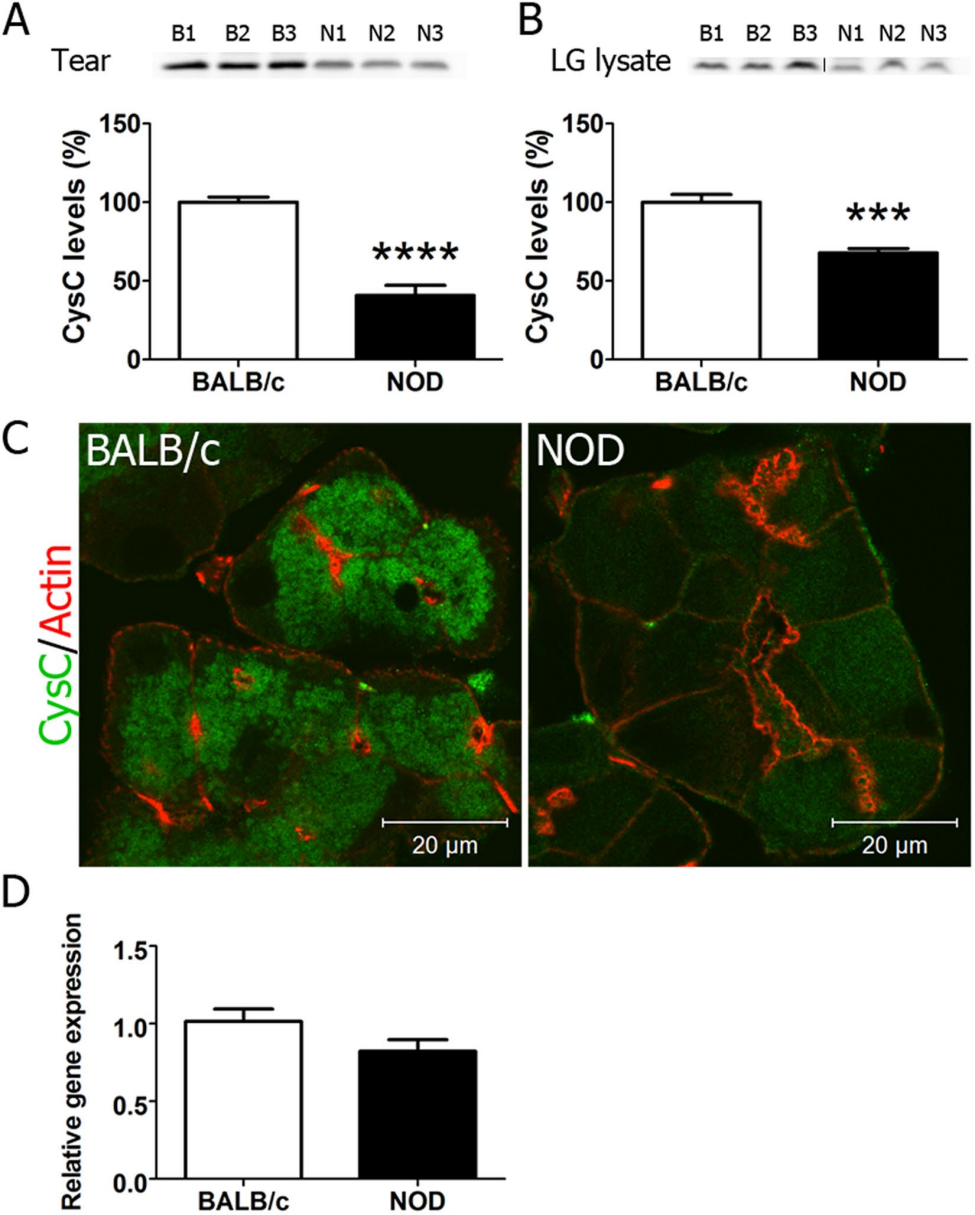
Expression of Cys C in Lacrimal glands and Lysates of Male NOD mice compared to BALB/c mice. Cys C concentration in (**A**) tears (n = 7 NOD and 7 BALB/c) and (**B**) LG lysates (n = 6 BALB/c and 6 NOD) from 12 week males as determined by Western blotting and quantification of Cys C signal by pixel intensity per µg protein. Representative blot is shown, Full length blot is shown in **(**Supplementary Fig. 1**)**. Values are presented as a percentage of those detected in BALB/c mouse samples, set at 100%. Error bars show SE; ***p < 0.001, ****p < 0.0001. (**C**) Immunofluorescence labeling of sections from LG of 12 week male BALB/c and NOD mouse LG for Cys C. Green, Cys C (labeled with Alexa-488 conjugated secondary antibody) and red, actin filaments labeled with rhodamine-phalloidin. (**D**) Relative gene expression of Cys C in LG acinar cells from 12 week male BALB/c (n = 6) and NOD (n = 6) mice isolated by laser capture microdissection.

We first analysed levels of Cys C in tears and LG of 12-week male healthy (BALB/c) and SS-model (NOD) mice. NOD males exhibit the autoimmune dacryoadenitis and reduced tear flow that constitute the principal ocular signs in SS patients^22^, and develop increased tear CTSS^4^, similar to SS patients. Western blotting revealed that Cys C was present in tears and LG lysates from NOD and BALB/c mice. The mean ± SE band intensity in NOD tears and NOD LG lysates was 41 ± 6.4% (p < 0.001) and 68 ± 2 0.8% (p < 0.0001), respectively of that in BALB/c mice. (Fig. 1A,B). Immunofluorescence analysis of Cys C confirmed its weaker expression in NOD mouse LG (Fig. 1C), although there was no difference in its gene expression (Fig. 1D), suggesting that the difference in its abundance could be due to increased degradation and/or decreased translation.

### Active CTSS enhances LF and Cys C degradation in tears of SS patients

**Figure 2:**
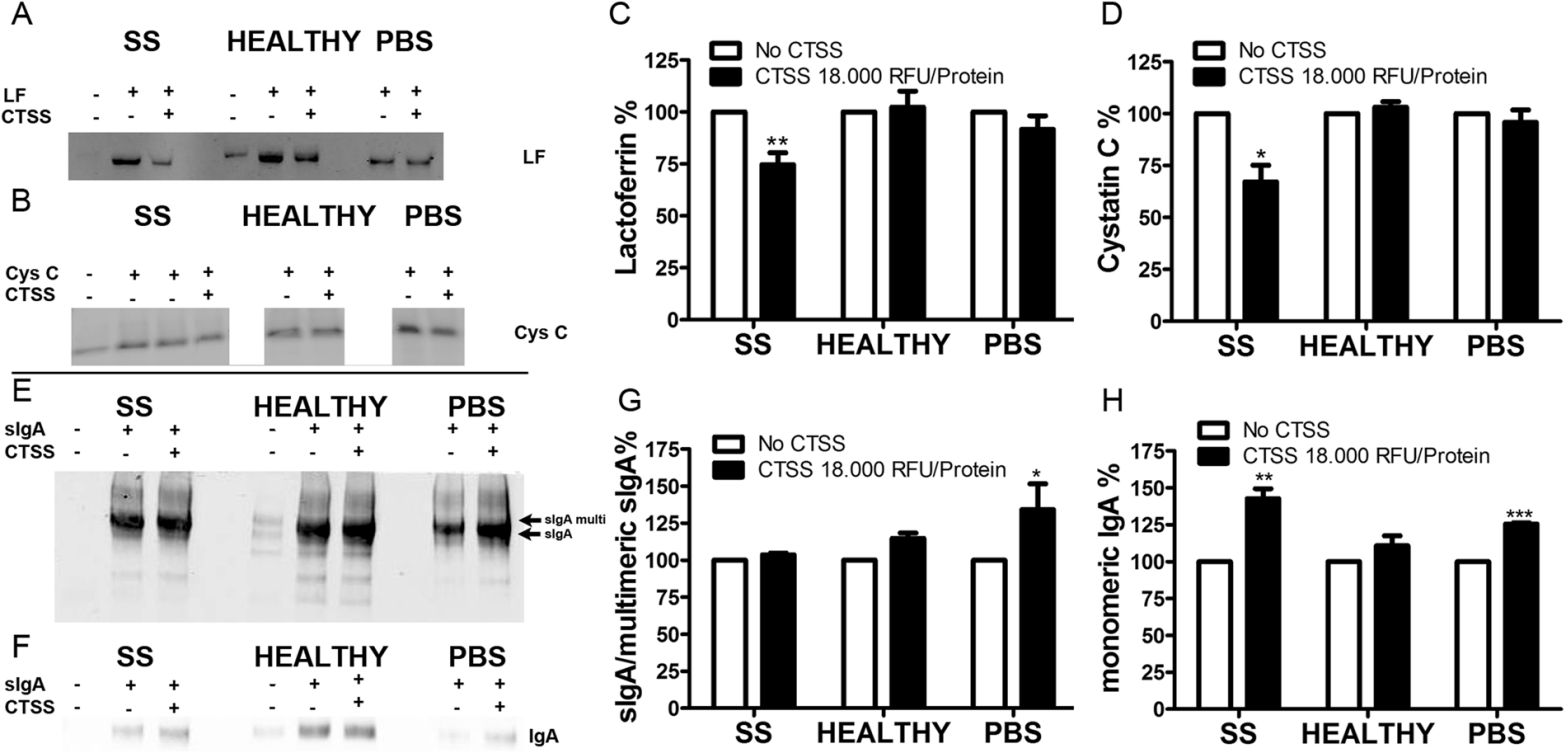
Degradation of LF, Cys C, and sIgA by CTSS occurs in tears from SS patients but not in tears from healthy controls. Tears obtained from SS patients and healthy controls were collected on Schirmer’s strips and were eluted in CTSS sample buffer. For LF degradation, tear samples were supplemented with 2.6 µg of recombinant LF only. For Cys C degradation, tear samples were supplemented with 2.5 µg Cys C and 2.6 µg LF. For sIgA degradation, tear samples were supplemented with 5 µg of purified sIgA. All supplemented tear samples were treated without or with recombinant CTSS equivalent to 18,000 RFU/10 mg tear protein for 4 hr at 37 °C. Equal amounts of recombinant LF, Cys C, and purified sIgA in PBS were used as controls. LF (**A**) and Cys C (**B**) loss by degradation was evaluated by Western blotting and densitometry as shown for LF in (**C**) and Cys C in (**D**). sIgA was blotted with both anti J-chain antibody (**E**) to detect multimeric IgA forms and with anti-IgA (**F**) antibody to detect monomeric IgA on the same gel. Densitometry is shown in (**G**) and (**H**), respectively. For quantitation, n = 6 for LF, n = 3 for Cys C and n = 4 for sIgA. Bars show SEM; *p < 0.05, **p < 0.01. Representative blots are shown. Full length blots are shown in Supplementary Figs 2 and 3.

With the significantly increased expression of CTSS in LG and tears in the NOD mouse that we have previously noted^4^, we hypothesised that one origin of decreased Cys C levels in tissue and tears could be enhanced degradation by increased CTSS activity. Further, we hypothesized that reductions in Cys C and possibly other tear protease inhibitors could create an environment capable of enhancing CTSS-mediated degradation, directly or indirectly, of other tear proteins. Since tears are not abundant, particularly in SS patients, pooled samples from several individuals was required to generate sufficient material to test this hypothesis. Operationally, this meant that SS and control tear samples were collected over a time period of as long as a month, with samples from individual subjects frozen at −80 °C during the collection period. Endogenous CTSS activity in tears is steadily lost over time, even when stored at −80 °C. This loss of CTSS activity over time notwithstanding, we hypothesized that any imbalance between CTSS and its endogenous inhibitors could still be demonstrated in tears that had been frozen. Accordingly, we supplemented SS and healthy control tears with equal amounts of recombinant active CTSS at an activity level at the higher end of the spectrum seen in SS patients^5^. We then measured the degradation of the tear proteins of interest (also supplemented as recombinant proteins) in the SS tear background versus the healthy control tear background. In SS patient tears supplemented with recombinant Cys C or LF and in the presence of CTSS, we observed enhanced degradation of both LF (Fig. 2A) and Cys C (Fig. 2B), with the mean ± SEM band intensities 74.6 ± 8.5% and 67.1 ± 8.0%, respectively, of the band intensity of the sample without added CTSS (Fig. 2C,D). We observed very little degradation of the recombinant LF and Cys C in PBS supplemented with CTSS, despite previous reports that CTSS can degrade LF^23^. These findings suggest that PBS may not provide an optimal environment for CTSS activity relative to SS tears under the conditions of this experiment and/or that CTSS-mediated degradation of LF and Cys C are largely mediated by the activation of other proteases present in SS tears activated by CTSS. In tears supplemented with purified sIgA and CTSS, no degradation of either J-chain or IgA itself was observed, but we detected accumulation of monomeric IgA in SS patient tears, possibly indicating degradation of multi/dimeric forms of IgA to monomeric IgA (Fig. 2E–H**)**.

### Validation of tear protein recovery from Schirmer’s strips and protein stability

Having demonstrated reduced Cys C in tears of NOD mice and shown that Cys C, LF and components of sIgA are susceptible to enhanced degradation mediated either directly or indirectly by CTSS in SS tears, we further hypothesised that the abundance of each of these three proteins might be reduced in SS tears. We also sought to determine whether tear levels of these proteins could increase the sensitivity and specificity in discriminating between SS and non-SS patients beyond that of tear CTSS activity alone.

Post-collection processing of tear samples may affect the detection and stability of some proteins^24^. To determine the extent of protein recovery from Schirmer’s strips, we collected tears from healthy volunteers (n = 3) with a glass capillary. Half of each sample was assayed immediately while the other half was added to a Schirmer’s strip and eluted. Recovery from Schirmer’s strips (mean ± SE) was 98.2 ± 1.5 for Cys C, 98.8 ± 0.5% for sIgA, and 96.3 ± 2.3% for LF. Optimal recovery conditions for CTSS were determined previously^5^.

To determine the stability of Cys C, sIgA, and LF at −80 °C, proteins eluted from Schirmer’s strips collected from SS and RA patients (n = 14 eyes total) were measured in the same samples within 4 hr of collection and after 1 month of storage at −80 °C. One-month recovery as a percentage of the values calculated within 4 hr of collection was (mean ± SE) 97.6 ± 8.4 for Cys C, 97.4 ± 4.9% for sIgA and 89.2 ± 3.6% for LF. Accordingly, tear CTSS activity was measured within 4 hr of tear collection, at which time no loss of activity occurred^5^, while sIgA, Cys C, and LF levels were measured in batches stored at −80 °C.

### Tear CTSS activity is increased while tear Cys C, sIgA, and LF concentrations are reduced in SS patients

We tested the potential of these four tear proteins to distinguish between SS patients from patients with non-SS autoimmune diseases or non-autoimmune DE. As the markers did not differ between patients with primary SS (n = 13) vs those with secondary SS (n = 20) **(**Supplementary Table 1**)**, the two groups were combined (n = 33).

**Figure 3:**
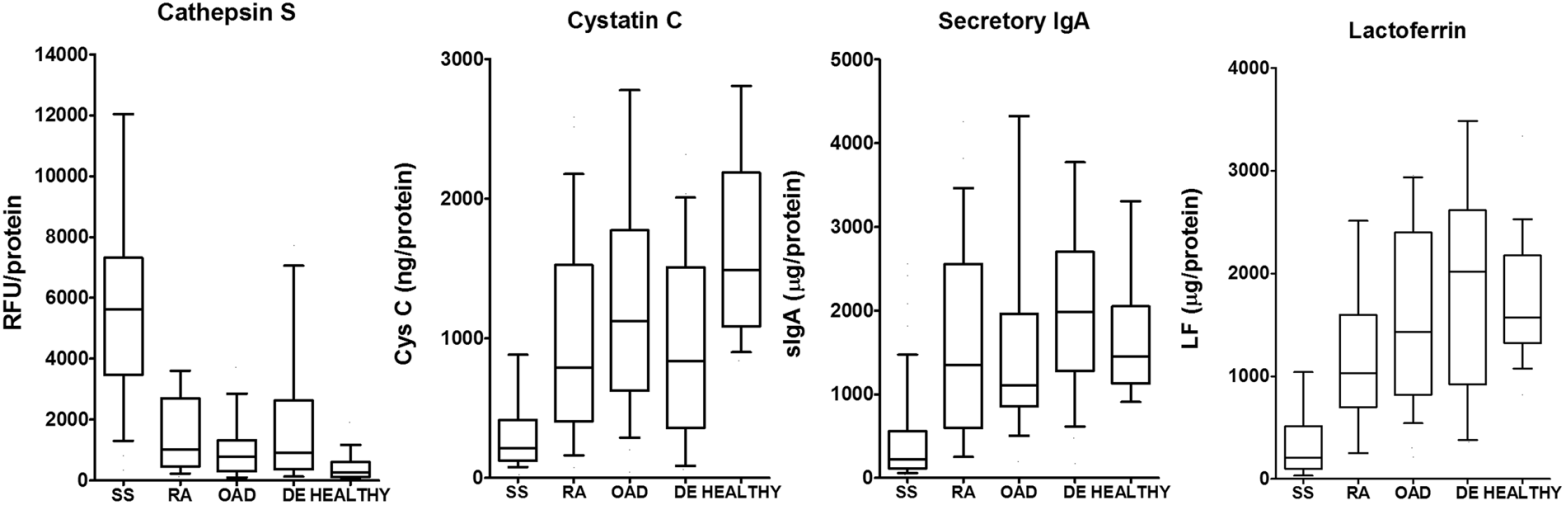
Cathepsin S (CTSS) activity, secretory IgA (sIgA) concentration, lactoferrin (LF) concentration, and Cystatin C (Cys C) concentration in tears from patients with SS, RA, other autoimmune diseases (OAD), non-autoimmune dry eye (DE) and healthy controls (HEALTHY). are plotted. Lines within the boxes represent median values; the boxes delineate the 25–75th percentiles; and the whiskers represent the 10–90th percentiles. Unit/protein indicates normalization to 10 mg of total tear protein.

**Figure 4:**
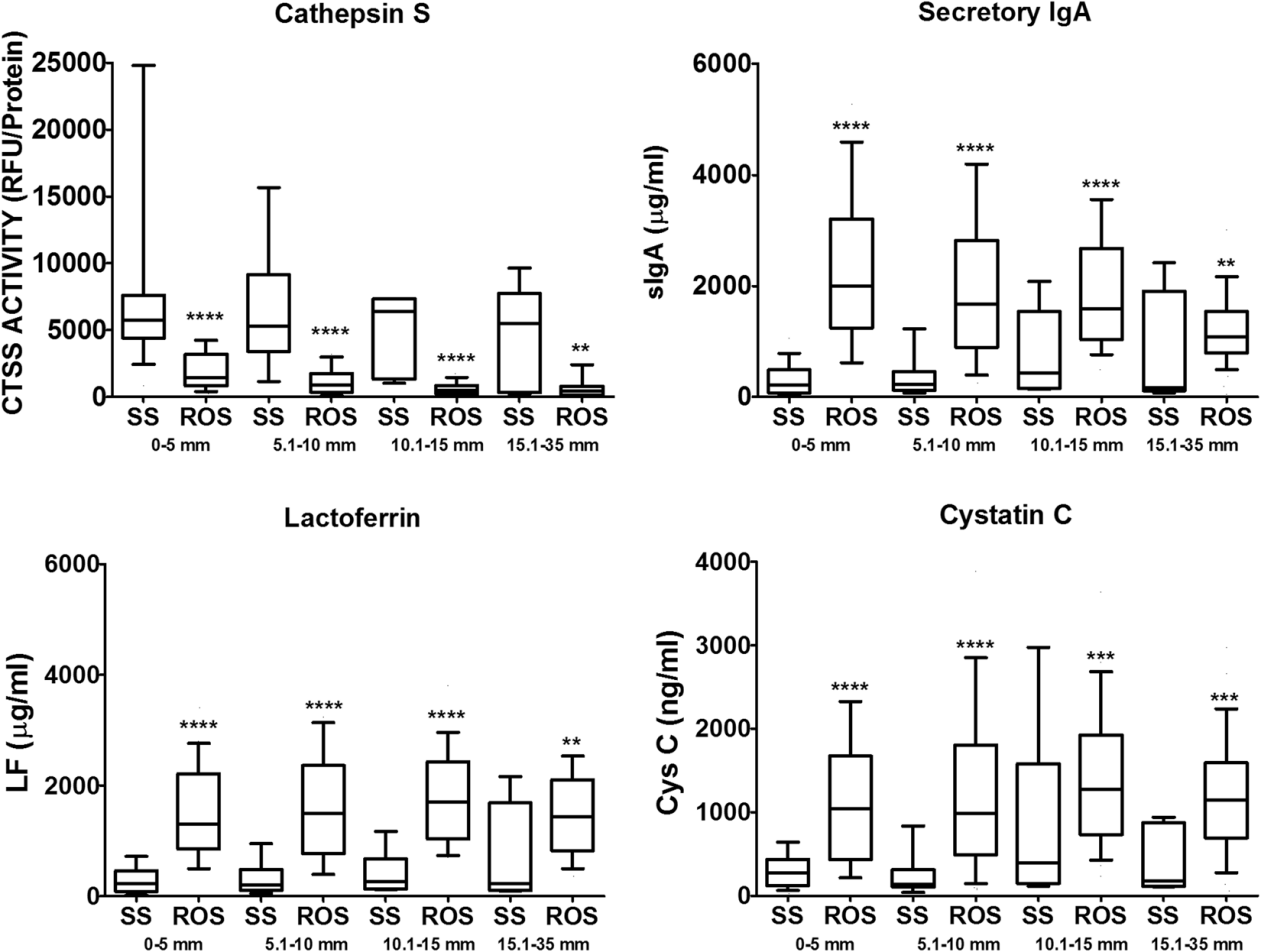
Relationship between each of CTSS, Cys C, sIgA and LF and Schirmer’s strip test values of each subject. Subjects were divided into 4 categories based on their Schirmer’s strip test values; 0–5 mm, 5.1–10 mm, 10.1–15 mm, and 15.1–35 mm. Statistical analysis was performed on values that were log transformed and adjusted to age, comparing SS patients to the rest of subjects (ROS) including RA, OAD, DE and healthy controls, within each category. Line represents median, box represents the 25–75th percentile, and whiskers represent the 10–90th percentile. *p < 0.05, **p < 0.01, ***p < 0.001, ****p < 0.0001. RFU; relative fluorescence units. Unit/protein indicates normalization to 10 mg of total tear protein.

In accord with our previous report^5^, tear CTSS activity was significantly higher in patients with SS than in patients with RA, other autoimmune diseases, non-autoimmune DE, or healthy controls. CTSS activity was elevated also in some non-autoimmune DE patients, with median CTSS activity being 3-fold (p = 0.003) higher than in healthy controls, compared to 21-fold (p < 0.0001) higher in SS patients compared to healthy controls. Tear sIgA, LF, and Cys C concentrations were each significantly reduced in SS patient tears compared to tear levels in all other groups (Fig. 3, Table 1), all comparisons were p < 0.0002. This was the case regardless of the volume of tears collected. In each range of Schirmer’s strip wetting (0–5 mm, 5.1–10 mm, 10.1–15 mm, > 15 mm), CTSS activity was significantly increased and Cys C, sIgA, and LF concentrations were significantly decreased in SS patients compared with tears from other subjects **(**Fig. 4**)**. Tear CTSS activity was inversely correlated with the concentrations of each of the other proteins (r ≤ −0.35; p < 0.0001; Supplementary Table 2). These three tear proteins also showed significant positive correlations (r ≥ 0.76, p < 0.0001) between each other. Of the candidate biomarkers, only CTSS was significantly correlated with age (r = 0.31, p < 0.0001).

**Table 1:** Activity of Cathepsin S and concentrations of secretory IgA (sIgA), Lactoferrin (LF), and Cystatin C in patients compared between diagnostic groups, n = 312 eyes, 156 subjects.

	SS^*^ (n = 33)	RA (n = 33)	Other (n = 31)	Dry Eye (n = 35)	Healthy (n = 24)
Age years (Mean ± SE)	55.8 ± 2.4	54.5 ± 2.4	49.0 ± 2.5	65.4 ± 2.3	45.7 ± 2.5
*p*	—	0.70^#^	0.10^#^	0.02^#^	0.02^#^
CTSS RFU/protein^**^ (Med ± SE)	5631 ± 937.9	1020 ± 205.5	786 ± 136.1	912.1 ± 399.2	267 ± 74.6
*p*	—	<0.0001^†^	<0.0001^†^	<0.0001^†^ 0.003^††^	<0.0001^†^
Cys C ng/protein** (Med ± SE)	211.7 ± 60.16	788.8 ± 121.3	1123 ± 117.5	834.2 ± 81.56	1490 ± 106.0
*P*	*—*	< 0.0001^†^	<0.0001^†^	0.0002^†^	<0.0001^†^
sIgA µg/protein^**^ (Med ± SE)	224 ± 97.0	1348 ± 147.0	1106 ± 163.2	1982 ± 152.6	1452 ± 121.9
*p*	*—*	<0.0001^†^	<0.0001^†^	<0.0001†	<0.0001^†^
LF µg/protein^**^ (Med ± SE)	207.1 ± 53.7	1031 ± 97.45	1428 ± 118.7	2018 ± 193.2	1572 ± 86.6
*p*	*—*	<0.0001^†^	<0.0001^†^	<0.0001†	<0.0001^†^

All data were normalized to the total protein concentration of the tear sample.

Groups were compared using generalized estimating equations to account for correlation between the eyes of each subject.

Within-subject correlation (between eyes) for comparison between SS, RA, OAD and healthy, CTSS r = 0.56; sIgA r = 0.74; LF r = 0.61; Cys C r = 0.62.

All models adjusted for age: p-value for association with age: CTSS p = 0.0005; sIgA p = 0.74; lactoferrin p = 0.86; cystatin C p = 0.98.

Overall p-value for group differences: CTSS p < 0.0001; sIgA p < 0.0001; LF p < 0.0001; Cys C p < 0.0001.

^*^Primary and secondary Sjögren’s syndrome combined.

^**^Indicates per 10 mg of total tear protein.

^#^ Overall p-value for group differences by ANOVA < 0.0001. P-values for difference from SS adjusted for multiple comparisons using Hochberg method.

^†^Statistical analysis is based on log transformed values, p is for comparison with SS.

^††^Statistical analysis is based on log transformed values, p is for comparison with Healthy.

SS = Sjögren’s Syndrome, RA = Rheumatoid Arthritis, Other = autoimmune disease other than SS or RA.

Assessed by logistic regression, each of the four biomarkers individually differentiated SS versus other autoimmune disease and non-autoimmune DE **(**Table 2**)**. However, multivariable analysis showed that neither Cys C, sIgA nor LF predicted SS in the autoimmune population when modelled with CTSS; CTSS remained a significant predictor of SS when modelled with each of the other proteins. In SS vs the DE population, CTSS similarly remained a predictor of SS when modelled with the other proteins. In addition, sIgA, LF and Cys C each remained predictors of SS when controlled for CTSS, but only LF remained significant when the three were modeled without CTSS (Table 2).

**Table 2:** Logistic regression of the four tear biomarkers singly and in combinations in Sjögren’s Syndrome patients^*^ versus the remaining autoimmune disease patients^**^ and in Sjögren’s patiens vs patients with dry eye not associated with autoimmune disease.

Model	*Autoimmune*		*Dry Eye*	
	Odds Ratio (95% CI)^***^	*P*	Odds Ratio (95%CI)^***^	*P*
***Biomarkers singly***				
CTSS	1.08 (1.05, 1.12)	<0.0001	1.03 (1.01, 1.05)	0.001
sIgA	0.83 (0.75, 0.92)	<0.0001	0.80 (0.72, 0.89)	<0.0001
LF	0.71 (0.61, 0.83)	<0.0001	0.73 (0.61, 0.87)	0.0004
Cys C	0.76 (0.66, 0.88)	0.0002	0.83 (0.73, 0.95)	0.007
**CTSS + sIgA**				
CTSS	1.07 (1.04, 1.11)	<0.0001	1.02 (1.00, 1.03)	0.037
sIgA	0.95 (0.87, 1.04)	0.26	0.82 (0.74, 0.92)	0.0003
**CTSS + LF**				
CTSS	1.06 (1.03, 1.10)	0.0004	1.03 (1.01, 1.04)	0.005
LF	0.87 (0.74, 1.03)	0.11	0.74 (0.63, 0.87)	0.0002
**CTSS + Cys C**				
CTSS	1.07 (1.04, 1.11)	<0.0001	1.03 (1.01, 1.04)	0.005
Cys C	0.91 (0.81, 1.03)	0.15	0.86 (0.74, 0.99)	0.033
**sIgA + LF + Cys C**				
sIgA	1.00 (0.89, 1.11)	0.94	0.88 (0.73, 1.06)	0.19
LF	0.75 (0.61, 0.92)	0.006	0.66 (0.46, 0.95)	0.026
Cys C	0.94 (0.82, 1.07)	0.36	1.48 (1.04, 2.11)	0.032
**CTSS + sIgA + LF**				
CTSS	1.06 (1.03, 1.10)	0.0004	1.03 (0.98, 1.03)	0.008
sIgA	1.00 (0.89, 1.11)	0.97	0.99 (0.86, 1.14)	0.89
LF	0.88 (0.71, 1.08)	0.22	0.75 (0.61, 0.93)	0.008

For each subject, the biomarker value used is the maximum value of the right eye or left eye for CTSS and minimum value of the right and left eye for Cys C, LF and sIgA.

^*^Primary and secondary Sjögren’s syndrome combined (n = 33).

^**^Rheumatoid Arthritis patients and patients with autoimmune diagnosis other than Rheumatoid Arthritis combined (n = 64).

^***^Odds ratios are expressed per 100 units of the biomarker.

**Figure 5:**
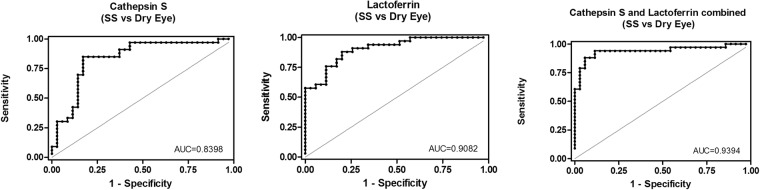
Receiving operating characteristic (ROC) for CTSS and LF in SS vs Dry Eye tears.

The area under the receiver operating characteristics (ROC) curve analysis further revealed high sensitivity and specificity for each of the four individual biomarkers in identifying SS vs other autoimmune patients and vs non-autoimmune DE (Table 3). All ROC were significantly greater than chance, with values being the highest for CTSS and the lowest for sIgA in the autoimmune population. In the non-autoimmune DE population, LF ROC was the highest and Cys C the lowest. Combining any of the three new candidate biomarkers with CTSS did not significantly improve the ROC values found with CTSS alone in the autoimmune population. However, for SS vs non-autoimmune DE, combining CTSS with LF significantly improved the ROC (from 0.840 to 0.939) **(**Fig. 5**)**.

**Table 3:** Comparison of receiver operating characteristics (ROC) between the four biomarkers, Cathepsin S (CTSS), secretory IgA (sIgA), Lactoferrin (LF) and Cystatin C (Cys C) as predictors of Sjögren’s Syndrome (SS)^*^.

	SS vs Total Autoimmune**		*SS vs Dry Eye*	
	ROC (95% CI)	*P* ^***^	ROC (95% CI)	*P* ^***^
CTSS	0.919 (0.851–0.986)		0.840 (0.740–0.940)	
sIgA	0.835 (0.740–0.982)		0.899 (0.819–0.978)	
LF	0.864 (0.787–0.939)		0.908 (0.841–0.975)	
Cys C	0.897 (0.602–0.855)		0.729 (0.602–0.855)	
CTSS + sIga	0.922 (0.856–0.989)	0.62	0.905 (0.828–0.983)	0.16
CTSS + LF	0.931 (0.870–0.993)	0.24	0.939 (0.878–1.000)	0.02 (0.18^****^)
CTSS + Cys C	0.961 (0.870–0.988)	0.11	0.857 (0.758–0.956)	0.63

^*^Primary and secondary Sjögren’s Syndrome combined.

^**^Rheumatoid Arthritis patients and patients with autoimmune diagnosis other than Rheumatoid Arthritis combined.

^***^p-value against CTSS alone.

^****^p-value against LF alone.

While these data suggest that measuring CTSS activity alone can sufficiently differentiate SS vs non-SS in both the autoimmune population and the non-autoimmune DE population, measuring LF in addition to CTSS may be of significant benefit in discriminating between SS patients and non-SS, non-autoimmune DE patients.

## Discussion

The etiology of SS is complex, with a multitude of environmental, genetic and hormonal factors implicated as driving forces. In recent years, CTSS, with its many functions linked to inflammation, has been linked to the etiology of many autoimmune diseases. The clinical efficacy of CTSS inhibitors is currently under investigation in inflammatory disorders including RA, SLE, plaque psoriasis, and, more recently, SS, both in animal disease models^25–27^ and in human clinical trials^28,29^. We have previously shown increased CTSS activity in tears of SS patients^5^, however, little is known about the basis for this increase or its impact on other tear components.

We show here that Cys C, an endogenous inhibitor and important regulator of CTSS activity, is produced and secreted by murine LG and that its levels are significantly reduced in the LG and tears of mice with autoimmune dacryoadenitis relative to levels in healthy mice. This finding of reduced tear Cys C was recapitulated in tears of SS patients compared to patients with other autoimmune diseases including RA and SLE as well as in patients with non-autoimmune DE. This reduction may contribute to the elevated CTSS activity seen in SS tears. Cox and coworkers have reported that only a small fraction (0.4–1.1%) of CTSS is enzymatically active in the sera of healthy donors and that attenuation of its activity is partly a result of the binding to Cys C^30^. A similar relationship between the two proteins may exist in other body fluids, including tears, as Cys C regulation of CTSS activity is effected through maintenance of a molar excess of Cys C. A shift in this balance occurs locally in other disease conditions associated with elevated CTSS activity including atherosclerosis and aortic aneurysm^16^. In patients with multiple sclerosis or asthma who are treated with glucocorticoids, a reduction of the serum ratio of CTSS to Cys C ratio is observed^31,32^, suggesting that the ratio may reflect the severity of inflammation. Along these lines, we have previously shown that LG and tear CTSS activity levels in male NOD mice are reduced by the immunomodulatory drug, rapamycin, administered i.v. or topically^33,34^, in parallel with reduced LG lymphocytic infiltration.

The current study further demonstrates that increased tear CTSS may also affect the quality of the tears in SS patients through direct or indirect degradation of other tear proteins. Accelerated degradation of LF and Cys C by CTSS was observed in SS patient tears when both active CTSS and recombinant LF or Cys C were added, suggesting a lack of protective factor(s) that might prevent CTSS-mediated proteolytic degradation in SS patient tears. It is possible that this protective factor is Cys C, but other proteases present in SS tears or a deficiency in other protease inhibitors may contribute. Cys C is known to be deactivated by degradation by MMP-2^35^ and Cathepsin D^36^. Cys C is also regulated by multiple transcriptional and post-translational factors^37^. For instance, pro-inflammatory cytokines active in SS including interleukin-6 and interferon-γ downregulate Cys C expression and secretion in immune cells^38^. Cys C activity can also be regulated conformationally; it may dimerise but loses the ability to bind its target proteases in this state^37^. Studies in transfected CHO cells showed that Cys C dimers can form transiently in specific cellular compartments in response to environmental factors like pH^39^.

We confirmed that Cys C, LF, and sIgA are all decreased in the tears of SS patients compared to tears from those with other rheumatic diseases, non-autoimmune DE, and healthy controls. The enhanced ability of CTSS to degrade these proteins in the biochemical background of SS tears (Fig. 2) is likely at least partially responsible for this reduction. However, a reduced secretory capacity of the acinar cells of the LG may also contribute to these changes. Indeed, it has been demonstrated that a principal secretory effector protein, Rab3D, is decreased in exocrine glands of SS patients, consistent with impaired production and/or release of some tear proteins^40,41^.

As an abundant tear protein produced by main and accessory LG^42^, the amount of LF in tears is indicative of general LG function. LF has been reported to be reduced in tears of patients with different forms of DE^43,44^, including mild evaporative DE^45^ and SS-associated DE^46,47^. Interestingly, in our study the non-autoimmune DE patients did not show a reduction in tear LF compared to healthy controls. It is possible that this phenomenon is due to the nature of most non-SS DE patients recruited at the Roski Eye Institute corneal clinic, a tertiary care clinic, who may represent a different spectrum of dry eye disease than populations in previous studies. Alternatively, differences in tear collection techniques, the use of glass capillaries versus elution from Schirmer’s strips or even the choice of an anesthetised versus an unanesthetised Schirmer’s test may also play a role^48^. It is important to note that analysis techniques have varied across studies from radial immunodiffusion to ELISA-based assays. The tear LF we report in our samples is also lower than that reported elsewhere, perhaps due in part to the lack of inclusion of protease inhibitors so that we can accurately measure CTSS activity.

Levels of tear sIgA in SS have surprisingly not been well-studied. Early observations in SS patient tears reported a decrease in total IgA rather than sIgA^43^. sIgA is a specific secretory protein released by proteolytic cleavage of the terminal domain of the polymeric immunoglobulin A receptor (secretory component) bound to IgA. Its recovery in mucosal fluids occurs after the IgA is bound and internalised from the serosal domain of the cells and transcytosed through the LG while bound to its receptor. The fact that there was only limited degradation of sIgA by CTSS suggests that its decrease may be due to reduced transcytotic trafficking and/or alterations in expression of receptors necessary for robust IgA antibody transport in the LG. The reduced recovery of tear LF may also reflect a possible decrease in LG secretion. Reduced secretion of both sIgA and LF are consistent with the functional quiescence and decreased secretory output associated with autoimmune dacryoadenitis^49^. The reduction of both sIgA, the principal component of mucosal defense, and LF, with robust antibacterial and anti-inflammatory properties^19,20^ and the ability to reduce oxidative stress^50^, suggests that SS patient tears may have a reduced ability to handle pathogens that may compromise ocular surface health.

The SS patients recruited for this study are typical of those found in “real-world” clinical practice, who were diagnosed by their rheumatologists based on clinical signs and on the presence of SSA autoantibodies but without labial or parotid biopsies or detailed ocular evaluation. A recent study by van Nimwegen *et al*.^51^ shows a strong agreement between diagnosis made by the treating rheumatologist and expert classification. The expert classification was based on anonymised case vignettes, containing a complete workup from a team of physicians including rheumatologists, oral and maxillofacial surgeons, pathologists and one ophthalmologist, and laboratory tests covering all of the items in each of the AECG, ACR, and ACR-EULAR criteria. This same study also investigated how individual ACR-EULAR items performed against the expert classification, showing that SSA seropositivity and clinical diagnosis, as used in our study, had excellent accuracy. Interestingly, tests measuring flow in unstimulated whole saliva and in tears (Schirmer’s test) showed much lower performance than expert classification with ROCs of 0.67 and 0.69, respectively, pointing to a great need for biomarkers of ocular involvement in SS.

Of note, the tear biomarkers identified in the present study showed equal ability to identify primary SS and secondary SS patients. This is of interest, since most established SS biomarkers focus on stratification of primary SS patients, whereas the majority of patients in “real-world” practice fall within the secondary SS category. Regardless, it will be important to further evaluate and validate these new tear tear biomarkers in larger cohorts of SS patients that are classified according to the ACR-EULAR criteria to fully determine their clinical usefulness and to identify their relationships to additional established SS biomarkers.

CTSS activity was elevated also in some non-autoimmune DE patients, although to a much lesser extent than in the SS patients. Since a complete SS workup was not performed on these DE patients, we cannot exclude that possibility that undiagnosed SS patients may be included in this group. It will be of importance to follow these DE patients with high CTSS activity longitudinally to see if they develop clinically-overt SS.

We show here that the elevated activity of CTSS found in LG lysates and tears of SS-model male NOD mice may be partially due to decreases in Cys C. These findings were extended to a clinical population, with the demonstration that tears of SS patients exhibit a similar imbalance between CTSS and Cys C, and suggesting that reduced levels of Cys C as well as possibly deficiencies in additional endogenous protease inhibitors may contribute to increased tear CTSS activity. This in turn may contribute to partial degradation of other tear proteins including Cys C itself, LF, and sIgA. Tear CTSS activity is reconfirmed here as a biomarker of SS in a cohort of patients distinct from that in our previous study. Moreover, combining tear CTSS and LF measurements may significantly improve the ability to distinguish SS patients from non-autoimmune DE patients. Investigation of additional tear proteins as potential substrates for tear CTSS may shed further insights into the unique mechanisms of ocular surface damage that affect SS patients.

## Materials and Methods

### General

Animal studies were conducted in accordance with the Guiding Principles for the Use of Animals in Research and approved by the University of Southern California (USC) Institutional Animal Care and Use Committee. Human studies were performed in accordance with the Declaration of Helsinki and approved by the Los Angeles County + University of Southern California Medical Center Institutional Review Board. All subjects provided written informed consent.

### Tear and tissue collection from mice

NOD mouse breeding pairs were purchased from Taconic (Rensselaer, NY) and bred in-house. Control BALB/c mice were obtained from Jackson Laboratories (Bar Harbor, ME). Male mice were used at 12 weeks, when the autoimmune dacryoadenitis characteristic of SS is established^4,22,52^. Tear collection by topical stimulation of the LG was as described previously^4,33^. Anesthetised mice were euthanised by cervical dislocation, and LGs were excised for use.

### Western blotting of Cys C in mouse tears and LG lysates

Tears from both eyes of each mouse were pooled, providing one sample per mouse with n = 7 per strain. LG lysates were prepared with a protease inhibitor cocktail described previously^53,54^. 10 µg of total tear protein and 40 µg of LG lysate protein from each mouse were resolved by electrophoresis on 4–20% PAGEr^TM^ EX gels (Lonza, Basel, Switzerland) and transferred to nitrocellulose membranes using an iBlot® 2 gel transfer device (Life Technologies, Carlsbad, CA). Western blotting was performed with a primary rabbit anti-Cys C polyclonal antibody (ab 109508, abcam, Cambridge UK) diluted 1:1000 and a secondary 700IRdyeDX conjugated goat anti-rabbit antibody Rockland, Limerick PA) diluted 1:2000. Membranes were scanned and analysed using an Odyssey infrared imaging system (Li-Cor, Lincoln, NE). Statistical analysis utilised a two-tailed Student’s t-test for independent samples, with p ≤ 0.05 as statistically significant.

### Tissue sectioning and immunofluorescence

LGs were fixed, sectioned, and prepared for immunofluorescence as described previously^54^. Sections were incubated with primary rabbit anti-Cys C antibody (ab109508, abcam) diluted 1:100, followed by anti-rabbit AlexaFluor488-conjugated secondary antibody (A21206, Molecular Probes, Eugene, OR) diluted 1:200. Samples were mounted with ProLong anti-fade mounting medium (Molecular Probes) and imaged by confocal fluorescence microscopy using an LSM 510 Meta NLO system (Carl Zeiss, Oberkochen, Germany).

### Laser capture microdissection and Cys C gene expression analysis

LGs were processed for laser capture microdissection, acinar cells collected, RNA isolated, and cDNA generated as described previously^54^. Quantitative PCR (qPCR) was conducted with the Cys C-specific primer: Mm00438347_m1 (Applied Biosystems, Foster City, CA) using an ABI 7900HT Fast Real-Time PCR System. Sdha (succinate dehydrogenase complex, subunit A) was used as the internal control. Reaction conditions and calculation methods were described previously^55^.

### Patient tear collection and elution of tear proteins

Topical anesthetic (Proparacaine 0.5%, Alcon, Fort Worth, TX) without fluorescein was applied to the eyes and tears were collected on Schirmer’s strips for 5 min. The strips were incubated in CTSS reaction buffer, obtained with the CTSS activity kit (BioVision, Milpitas, CA), and centrifuged at 6700 RCF for 15 sec in an Eppendorf (Hamburg, Germany) minispin centrifuge to elute proteins.

### Effect of active recombinant CTSS on LF, Cys C and sIgA in tears

Eluted proteins from tears of both eyes of each healthy control subject were pooled. Due to low sample volume, samples from both eyes of pairs of SS patients collected sequentially were pooled for each replicate sample, thus samples from both eyes of 12 patients gave n = 6 for LF and samples from 6 patients gave n = 3 for Cys C. For LF degradation analysis, 2.6 µg recombinant LF only (Novatein, Woburn, MA) was supplemented into tear samples. For Cys C degradation analysis, 2.5 µg purified human Cys C (Calbiochem, Burlington, MA) was supplemented to tear samples which also contained recombinant LF as above. In separate samples from both healthy controls (n = 4, single subjects) and SS patients (n = 4, single patients), 5 µg of purified human secretory IgA (MP Biomedicals Santa Ana CA) was added. To allow for concurrent analysis of multiple samples side by side and inclusion of appropriate controls, tear samples from several SS patients and healthy controls were needed, requiring collection over a period of weeks as sufficient SS patients were recruited. Tear samples were stored frozen at −80 °C for up to a month prior to experiments as sufficient material was collected for the assays. These conditions allow loss of endogenous CTSS enzymatic activity, but we hypothesized that any imbalance that might exist between CTSS and its endogenous inhibitors would still exist in the frozen tears. To probe this, samples were supplemented without or with recombinant CTSS (BioVision), corresponding to an activity level found in the 90–95th percentile of SS patient tears in our previous study cohort^5^ (18,000 RFU/10 mg tear protein) for 4 hr at 37 °C. Total protein concentration of each sample was measured using the Bio-Rad (Hercules, CA) protein assay. Equal amounts of total protein from each sample were resolved either in the presence of β-mercaptoethanol (reducing conditions), to optimize separation for Cys C and LF, or under non-reducing conditions, to avoid subunit separation for sIgA, on 4–20% PAGEr^TM^ EX gels. Gels were transferred to nitrocellulose membranes which were blocked with blocking buffer (Rockland) before incubation with anti-LF (SC 101487, Santa Cruz Biotechnology, Santa Cruz CA) diluted 1:1000 or anti-Cys C (ab 109508, Abcam) diluted 1:1000 mouse monoclonal antibodies or with anti-J-chain (MA180527, Thermo Scientific, Waltham MA) diluted 1:500 or anti-IgA (MAB4787–100, R&D systems, Minneapoliis, MN) diluted 1:2000 mouse or rabbit antibodies, respectively, followed by incubations with appropriate secondary antibodies labeled with IRdye 700DX or 800DX (Rockland) diluted 1:5000. Band intensity was measured by densitometry and significance was determined by an unpaired student’s t-test, with p ≤ 0.05 considered statistically significant.

### Human subjects

97 female patients (13 with primary SS; 20 with secondary SS; 33 with RA; and 31 with other systemic inflammatory disease, including SLE, psoriatic arthritis, Crohn’s disease, ankylosing spondylitis, and sarcoidosis) over the age of 25 were recruited from the LAC + USC Medical Center or Keck Medicine of USC Rheumatology clinics. Diagnosis was assigned based on Ro/SSA autoantigen seropositivity and the clinical judgment of the attending physician. 35 female patients with non-autoimmune DE disease, including both evaporative and aqueous-deficient DE, were recruited from the USC Roski Eye Institute cornea clinic. DE patient diagnosis was based on slit lamp examination evaluating punctate epithelial erosions (PEE, on a scale where 0 is the absence of PEE and 4 is severe PEE), tear film quality, and volume. Thirty of our 35 patients had a mild PEE score (≤1) with an average across our population of 1.02. We could not perform fluorescein or lissamine green staining at the time of tear collection, since trace amounts of these fluorescent stains in tears would interfere with the CTSS enzyme activity assay. Patients who had undergone eye surgery within six months of the tear collection were excluded. Treatment regimens for each patient were recorded at the time of recruitment but were not a basis for inclusion or exclusion (Supplementary Table 3**)**. Patients who participated in our previous study of CTSS activity in SS patients^5^ were excluded from the present study. Healthy female control subjects (n = 24) from LAC + USC Medical Center or USC School of Pharmacy staff were selected to match the age and ethnicity of the patient population. Control subjects were not taking any medications and did not have a history of rheumatic or DE disease.

### Tear protein quantification

CTSS activity was determined with the CTSS activity kit (BioVision) according to the manufacturer’s instructions. Activity values were normalised to total protein concentration as described previously^5^. sIgA, LF, and Cys C levels in tears eluted from Schirmer’s strips were quantified using the sIgA ImmuChrom ELISA Kit (Eagle Biosciences, Amhurst NH), lactoferrin (HLF2) human ELISA Kit (Abcam), and Cys C quantikine human Cys C ELISA kit (R&D Systems), respectively. Final tear dilutions were 1:5,000 for sIgA, 1:60,000 for LF, and 1:50 for Cys C.

### Statistical analysis

Results for each of the four candidate biomarkers were natural log-transformed to achieve normality for statistical analyses involving correlations and comparisons of means. As tear biomarkers were measured from each eye, subjects contributed two values for each marker. To account for the correlated data, statistical comparisons of biomarker means among study groups used generalised estimating equations for correlated outcomes; an identity link function and an exchangeable correlation structure were used. Group comparisons were adjusted for age. To evaluate the ability of each biomarker to discriminate between subjects with and without SS, logistic regression analyses were performed; each subject contributed one observation, using the maximum CTSS and minimum Cys C, sIgA and LF values across the two eyes. Each biomarker was first tested individually in a logistic regression model. Because we had previously shown a strong association of elevated CTSS in SS patients compared to other autoimmune disease and healthy controls^5^, multivariable logistic regression models included CTSS and tested the added contribution of sIgA, LF, and CysC. Logistic regression results are summarised as odds ratios, with 95% confidence intervals. As a measure of group differentiation, area under the receiver operating characteristic (ROC) curves were computed and tested for differences among different models. ROC curves were compared for: (1) SS vs. autoimmune disease (RA, and other autoimmune groups combined, n = 64); and (2) SS vs. non-autoimmune DE (n = 35). For each subject the biomarker value used is the maximum value of the right eye or left eye for CTSS and the minimum value of the right eye and left eye for Cys C, LF and sIgA. 

### Data availability

The datasets generated during the current study are available from the corresponding author on reasonable request.

## Electronic supplementary material

Supplementary Material
