# Supplementary material for: Increased Cathepsin S activity associated with decreased protease inhibitory capacity contributes to altered tear proteins in Sjögren’s Syndrome patients
Source: Sci Rep. 2018 Jul 23;8:11044. doi: 10.1038/s41598-018-29411-9 (PMC6056496; doi:10.1038/s41598-018-29411-9)
Supplement: Supplementary file 1 — Supplementary Material [file 41598_2018_29411_MOESM1_ESM.pdf]

Increased Cathepsin S activity associated with decreased protease inhibitory capacity contributes to altered tear proteins in Sjögren's Syndrome patients

Maria C. Edman PhD[1], Srikanth R. Janga MS[1], Zhen Meng PhD[2,], Mercy Bechtold MD[3], Alexander F. Chen MD[3], Chongiin Kim[3], Luke Naman[3], Arunava Sarma[3], Neha Teekappanavar[3] Alice Y. Kim MD[3], Sara Madrigal MA[4],Simranjit Singh MD[4], Elizabeth Ortiz MD[4], Stratos Christianakis MD[4], Daniel G. Arkfeld, MD[4], Wendy J. Mack PhD[5], Martin Heur MD, PhD[1], William Stohl MD, PhD[4,6] and *Sarah F. Hamm-Alvarez, PhD[1,2]

[1] Department of Ophthalmology, USC Roski Eye Institute, Keck School of Medicine, University of Southern California, Los Angeles, CA

[2] Department of Pharmacology and Pharmaceutical Sciences, School of Pharmacy, Los Angeles, University of Southern California, Los Angeles, CA

[3] Keck School of Medicine, University of Southern California, Los Angeles, CA

[4] Division of Rheumatology, Department of Medicine, Keck School of Medicine, University of Southern California, Los Angeles, CA

[5] Department of Preventive Medicine, Keck School of Medicine, University of Southern California, Los Angeles CA

[6] Division of Rheumatology, Department of Medicine, Los Angeles County + University of Southern California Medical Center, Los Angeles, CA

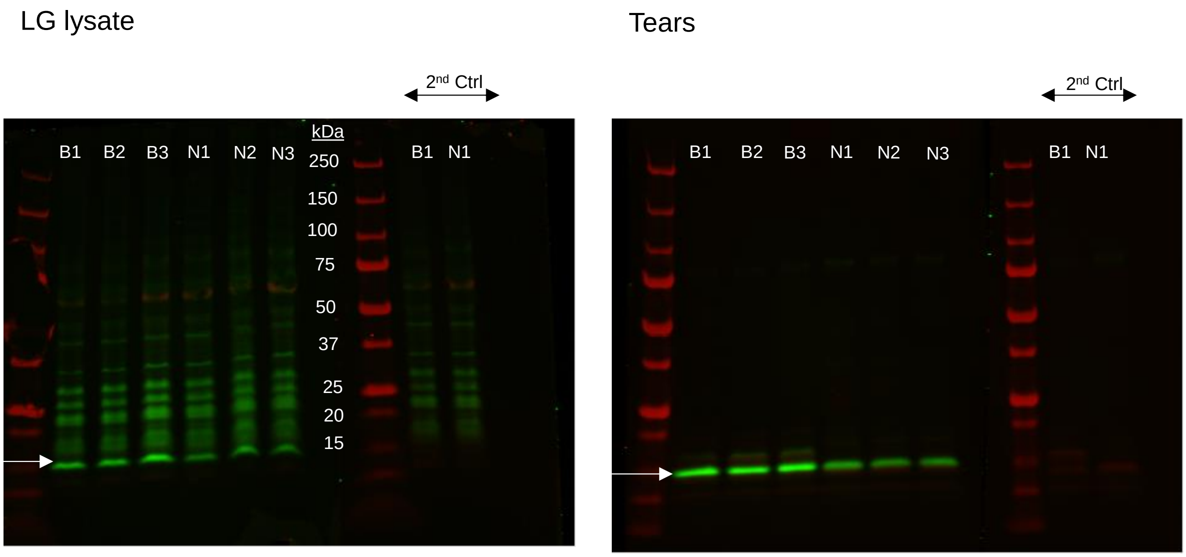

Supplementary Figure S1. Full length Western Blots and secondary controls of Cystatin C from 12 week male BALB/c or NOD mice shown as cropped blots in Figure 1A and B. Western blotting was performed with a primary rabbit anti-Cys C polyclonal antibody (Abcam) and a secondary 700IRdyeDX conjugated goat anti-rabbit antibody (Rockland). Membranes were scanned using an Odyssey infrared imaging system (Li-Cor), The molecular weight ladder is showed in the IR 700 channel (Red) and Cystatin C bands in the IR 800 channel (Green). Secondary antibody only controls from one sample of each stain are shown to the right.

Cystatin C

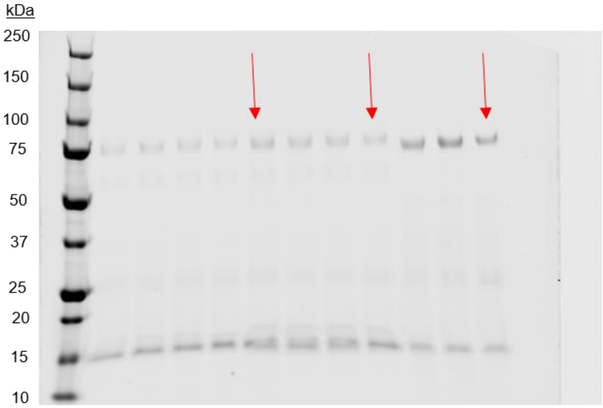

lactoferrin

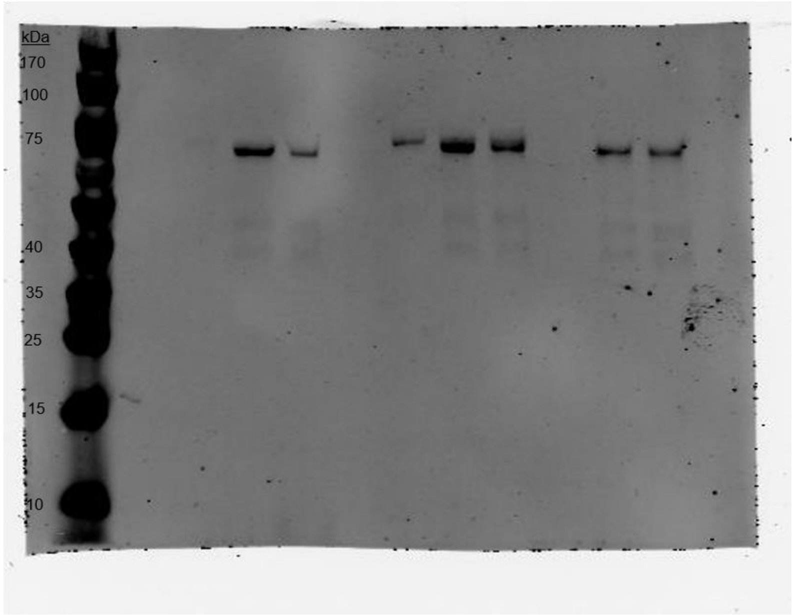

Supplementary Figure S2. Full length Western blots of Cystatin C (top) and Lactoferrin (bottom) shown as cropped blots in Figure 2. In the Cystatin C blot, lanes marked with a red arrow in the Cystatin blot were spiked with active CTSS at an activity level corresponding to 54,000 RFU/10mg of tear protein (3 times the activity levels found in SS tears. However, as this experimental condition is not physiologically relevant, we choose not to show or discuss this condition in the main paper. Tear samples in Cys C blots only were also supplemented with Lactoferrin (2.5 μg), and due to substantial secondary antibody binding to Lactoferrin, a Lactoferrin band can also be observed at 75 kD,

IgA

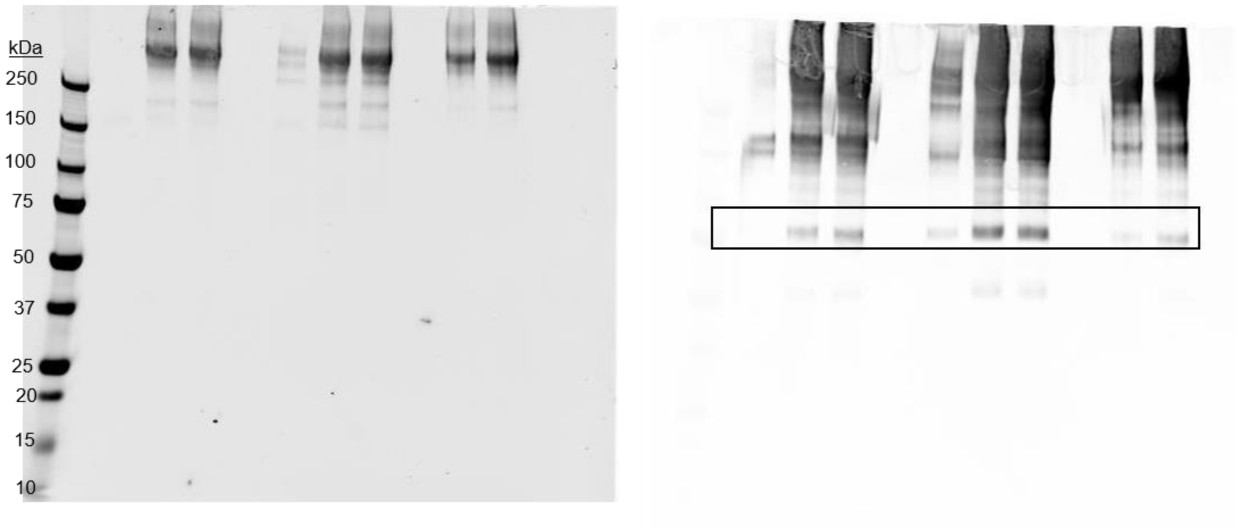

Supplementary Figure 2. Full length Western blots of J-chain (left) and IgA (right), shown as cropped blots in Figure 2. J-Chain and IgA blots were performed on the same gel but imaged in the IR700 channel or IR800 channels, respectively. Box indicates shown monomeric IgA band.

Supplementary Table 1. Activity of Cathepsin S and concentrations of secretory IgA (sIgA), Lactoferrin (LF), and Cystatin C in patients with primary SS (pSS) vs patients with secondary SS(sSS) (n=66 eyes, 33 subjects).

	pSS (n=13)	sSS (n=20)
Age years (Mean±SE)	54.7±3.7	56.6±2.1
p	-	0.24
CTSS RFU/protein* (Med±SE)	8259.3±2045.0	6074.3±737.9
p	-	0.98
Cys C ng/protein* (Med±SE)	480.0±110.3	554.2±141.6
p	-	0.83
sIgA µg/protein* (Med±SE)	473.1±99.2	312.4±54.2
p	-	0.21
LF µg/protein* (Med±SE)	336.6±55.6	394.8±91.4
p	-	0.45

All data were normalized to the total protein concentration of the tear sample.
Groups were compared using generalized estimating equations to account for correlation between the eyes of each subject.
Within-subject correlation (between eyes): CTSS r=0.53; sIgA r=0.95; LF r=0.89; Cys C C r=0.78
P-value for association with age: CTSS p=0.02; sIgA p=0.83; LF p=0.50; Cys C p=0.93
* indicates per 10 mg of total tear protein

Supplementary Table 2: Spearman Correlations (r) between the biomarkers, Cathepsin S (CTSS), secretory IgA (sIgA), Lactoferrin (LF) and Cystatin C (Cys C), age and Schirmer's values.

	Age	*Schirmer's*	*CTSS*	*sIgA*	*LF*
CTSS	0.28[*]<br>p<0.0001	-0.48<br>p<0.0001<br>*-0.048*[**]<br>*p=0.0005*			
sIgA	0.09<br>p=0.12	0.03<br>p=0.54<br>*-0.31*<br>*p=0.035*	-0.35<br>p<0.0001		
LF	-0.02<br>p=0.79	0.23<br>p<0.0001<br>*0.32*<br>*P=0.03*	-0.47<br>p<0.0001<br>*-0.41*<br>*p=0.0034*	0.76<br>p<0.0001	
Cys C	-0.11<br>p=0.05	0.21<br>p<0.0003	-0.42<br>p<0.0001	0.66<br>p<0.0001	0.72<br>p<0.0001

[*] Values in plain text indicate correlation in the total population, n=312 eyes,
[**] Values in *Italic* text indicate correlation in healthy controls only, n=48 eyes, only significant correlations are shown.

Supplementary Table 3: Distribution of the major drug classes used for treatment of autoimmune disease patients by diagnosis.

**Systemic drugs**	**SS**	**RA**	**Other**	**DE**
DMARD or Cytotoxics (only)	39 %	39 %	29 %	11%
Biologic (only)	0 %	10 %	10 %	0%
Steroid (only)	7 %	0 %	3 %	0%
DMARD +Biologic	11 %	13 %	10 %	0%
DMARD + steroid	18 %	19 %	16 %	0%
Biologic + steroid	0 %	0 %	3 %	0%
DMARD + Biologic + steroid	7 %	13 %	6 %	0%
**Ocular topical drugs**				
Restasis	18 %	0 %	0 %	27%
Xiidra	0%	0%	0%	2%
Ocular hypertension drugs	0%	0%	0%	5%
Steroid eye drops	0%	0%	0%	8%
OTC eye drop	39%	16%	3%	39%
